# Contribution of radixin and ezrin to the maintenance of hepatocytes' excretory function in health and disease

**DOI:** 10.1016/j.heliyon.2023.e21009

**Published:** 2023-10-18

**Authors:** Friederike Dellbrügge, Lena D. Jesse, Anna Medyukhina, Na Liu, Sophie Neugebauer, Markus Freißmuth, Stephanie Höppener, Marc T. Figge, Helen Morrison, Lars B. Riecken, Adrian T. Press

**Affiliations:** a Department of Anesthesiology and Intensive Care Medicine, Jena University Hospital, Am Klinikum 1, 07740, Jena, Germany; b Center for Sepsis Control and Care, Jena University Hospital, Am Klinikum 1, 07740, Jena, Germany; c Research Group Applied Systems Biology, Leibniz Institute for Natural Product Research and Infection Biology - Hans Knoell Institute, Beutenbergstraße 11a, 07745, Jena, Germany; d Department of Clinical Chemistry and Laboratory Diagnostics, Jena University Hospital, Am Klinikum 1, 07740, Jena, Germany; e Laboratory of Organic and Macromolecular Chemistry (IOMC), Friedrich-Schiller University, Fürstengraben 1, 07737, Jena, Germany; f Faculty of Biological Sciences, Friedrich-Schiller University, Fürstengraben 1, 07737, Jena, Germany; g Leibniz Institute on Aging, Beutenbergstraße 11, 07745, Jena, Germany; h Medical Faculty, Friedrich-Schiller University, Fürstengraben 1, 07737, Jena, Germany

**Keywords:** Liver, Cholestasis, ERM proteins, MRP2

## Abstract

**Background & aims:**

Excretory liver failure is frequently associated with poor prognosis in critically ill patients. It is characterized by the loss of canalicular membrane export pumps at the hepatocyte membrane. The membrane export pump Multidrug resistant-associated protein (MRP) 2 is pivotal in hepatocytes for brushed membrane morphology and transport of various metabolites. In addition, MRP2 anchoring proteins of the Ezrin/Radixin/Moesin (ERM) family are crucial for the correct MRP2 location, integration, and function in different tissues. In hepatocytes, altered ERM signaling is elementary for developing excretory liver failure.

**Methods:**

Polarized human HepaRG cells, primary human hepatocytes, and hepatocyte-specific Ezrin knockout mice are employed to investigate ERM expression and function in health and the bile duct ligation model of obstructive cholestasis.

**Results:**

ERM-scaffolding protein Ezrin has no relevant function in maintaining the canalicular structure in hepatocytes during health and disease.

**Conclusions:**

Homeostasis of the canalicular pole in hepatocytes is maintained exclusively by Radixin but not Ezrin, and Radixin dysfunction promotes cholestasis.

## Graphical abstract

**Figure:**
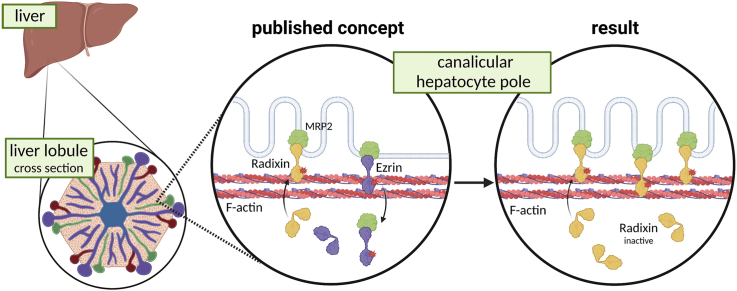

## Abbreviations

ABCC: ATP binding cassette subfamily C member
ALAT: alanine aminotransferase
AlbCre^ki/ki^: B6.Cg-Tg(Alb-cre)21Mgn/J
ASAT: aspartate aminotransferase
BDL.: bile duct ligation
BSA: bovine serum albumin
BSEP: bile salt export pump
BW: body weight
CA: cholic acid
CDCA: chenodeoxycholic acid
DCA: deoxycholic acid
ERM: Ezrin/Radixin/Moesin
Ezr^fl/fl^: B6-TG(Ezr-loxp-deltaβ-geo)
Ezr^ko/ko^: hepatocyte-specific Ezrin knockout
FBS: Fetal bovine serum
FERM: N-terminal band 4.1 protein, Ezrin, Radixin and Moesin
GM-CSF: Granulocyte-macrophage colony-stimulating factor
IFN: Interferon
IL.: Interleukin
LCA: lithocholic acid
MCP: Monocyte chemoattractant protein
MRP2: Multidrug resistant-associated protein 2
NPC: non-parenchymal cells
PBS: Phosphate Buffered Saline
PC: principal component
PCA: principal component analysis
pERM: EzrinT567, RadixinT564, and MoesinT558
PHH: Primary human hepatocytes
Rdx: Radixin
T: Threonine
TBS: Tris Buffered Saline
TNF: Tumor necrosis factor
UDCA: ursodeoxycholic acid

## 1 Introduction

Cholestasis characterizes a decrease in bile excretion or bile flow. As a factor of excretory liver failure, it accrues in many diseases - including infectious liver failure, intoxication, or tumors - where it is strongly associated with poor prognosis and mortality [[1], [2], [3], [4]]. Distinct changes in the function and morphology of the hepatocellular canalicular brush border have been described in most cholestatic diseases [[5], [6], [7]]. At the hepatocellular brush border, the integration of ATP-dependent transporters, especially the Multidrug resistance-associated protein 2 (MRP2), links this unique membrane morphology with its function [8,9]. Dislocation of MRP2 from the canalicular membrane, accompanied by structural changes of the brush border, is a hallmark of cholestatic diseases [6,10].

The Ezrin/Radixin/Moesin (ERM) protein family anchors transporters at the canalicular membrane [5,11]. In different tissues, ERM proteins regulate the trafficking and localization of various integral membrane receptors and transporters. In most polar cells, they are found in apical membrane structures [12,13]. As part of protein complexes, ERM proteins function as anchoring and signaling molecules through direct [[14], [15], [16]] or indirect binding [17,18] to integral membrane proteins. This interaction is mediated by ERM's highly conserved N-terminal band 4.1 protein, Ezrin, Radixin and Moesin (FERM) domain [19]; while the C-terminal region, likewise highly conserved, contains the actin-binding site and a consensus phosphorylation site required for activation [20,21]. Phosphorylated Ezrin Threonine (T)567, Radixin T564, and Moesin T558 change into a linearized conformation, which unmasks C- and N-terminal binding sites otherwise masked by intramolecular binding [22]. Upon activation, the C-terminal domain binds to actin, stabilizing the localization of their N-terminal binding partners in membrane compartments [23].

In liver tissue, all three isoforms are found in the different cell types. Moesin expression is restricted to hepatic stellate cells, where it links the actin cytoskeleton to the cell membrane, a function crucial for cell migration [24]. Cholangiocytes express Ezrin at the apical membrane [25], while Ezrin- and Radixin-mediated signaling has been described in hepatocytes. Radixin expression in hepatocytes has been well characterized in knockdown and knockout experiments, but the function and expression of Ezrin remain controversial [5,25,26]. Hepatocytes of Radixin knockout mice and rat primary hepatocytes after Radixin knockdown lack the characteristic canalicular brush border and fail to localize and maintain cell transporter pumps, such as MRP2 or bile salt export pump (BSEP) - both essential in eliminating endo- and xenobiotics - at the canalicular membrane [27,28]. Disturbed colocalization of canalicular Radixin and MRP2 in combination with intracellular retrieval of MRP2, has been observed in patients suffering from cholestatic diseases, including drug-induced and obstructive cholestasis [5]. Those findings were supported by pre-clinical experiments in rat liver and isolated rat hepatocytes, which showed that the interaction of Mrp2 and Radixin depends on Radixin T564 activating phosphorylation and that Radixin dephosphorylation and Mrp2 intracellular retrieval is associated with a decrease in bile flow [11,29].

The strong effects of Radixin localization and expression on the canalicular membrane structure in humans and rodents lead to the conclusion that Radixin is the predominant ERM protein in hepatocytes. In cholestasis, Radixin dephosphorylation promotes MRP2 translocation and structural membrane remodeling.

An exciting observation was the mechanism whereby Ezrin localized at the canalicular membrane of hepatocytes, contrary to Radixin, stimulates the internalization and degradation of canalicular MRP2 upon its activation [26]. Existing Ezrin knockout and knockdown studies have been inconclusive regarding Ezrin function and expression in hepatocytes. Ezrin null-mice (Vil2^−/−^) die before weaning from a defect of villus morphogenesis in the intestines, indicating the importance of the Ezrin isoform in membrane shaping [30]. Another study utilized an Ezrin knockdown mouse (Vil2^KD/KD^) with Ezrin expression reduced to 5 % of wild-type mice. The authors presented severe growth retardation and frequent juvenile death of Vil2^KD/KD^ mice. Some animals reached adulthood but suffered from achlorhydria [31], hypophosphatemia [32] and intrahepatic cholestasis [25]. Following those reports, Hatano et al. attributed the intrahepatic cholestasis of Vil2^KD/KD^ mice to ion-exchange pump defects and misguided transporter trafficking in cholangiocytes [25,33]. Considering the Ezrin function in hepatocytes described by others, additional hepatocellular contribution to the molecular basis of cholestasis in Vil2^KD/KD^ mice still has not been conceptualized.

To further examine ERM proteins' crucial role in hepatocellular membrane constitution, particularly during cholestasis, our study uses liver-specific Ezrin knockout mice to enlighten Ezrin expression and function in hepatocytes.

## 2 Results

### 2.1 ERM expression in liver tissue, primary hepatocytes, and HepaRG cells

**Fig. 1:**
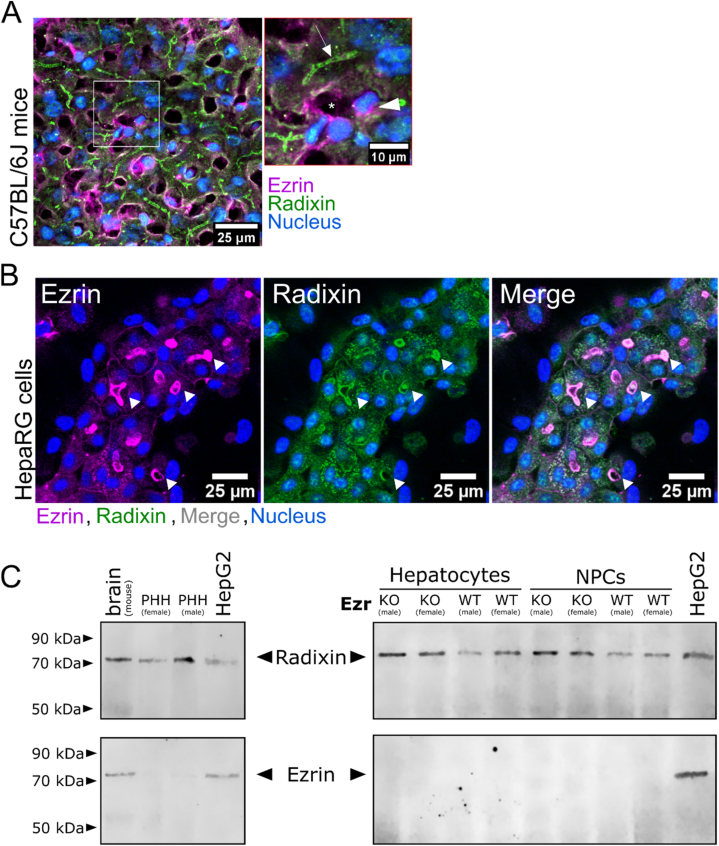
**Expression of Ezrin/Radixin/Moesin in murine liver and HepaRG cells and primary hepatocytes** (**A**) Murine liver tissue stained for Ezrin (magenta), Radixin (green), and nucleus (blue). Arrow depicts an exemplary bile canaliculus exclusively stained for Radixin. Triangle points towards an immune cell expressing Ezrin. The asterisk marks a sinusoid. Representative image from three stained mice. (**B**) Staining of the nucleus (blue), Ezrin (magenta), and Radixin (green) in HepaRG cells. The overlay of both channels appears grey. Triangles point out some canalicular-like structures formed by HepaRG cells. Three individually differentiated batches of HepaRG cells resulted in similar staining. (**C**) Western blot depicting Ezrin and Radixin expression of the immortal human cell line HepG2 and primary human hepatocytes (PHH) from 20 male or female donor pools. Ezrin and Radixin expression in mice were analyzed in primary hepatocytes and non-parenchymal cells (NPCs) from donor pools of 3 mice per sex and genotype (C57BL/6 (WT) or hepatocyte-specific Ezrin knockout (KO)). For the corresponding electrophoresis gels and the uncropped membranes, see Supplementary Fig. 1.

We examined Ezrin and Radixin distribution in liver tissue and cells. Echoing results from previous studies, Radixin was detected in hepatocytes of murine liver tissue. The mid-line pattern of the Radixin staining in hepatocytes indicates its localization at the apical canalicular pole. Further, Ezrin stained the liver's sinusoidal-located cells but was not detected in hepatocytes (Fig. 1A).

In contrast, human HepaRG cells, an immortal hepatic cell line forming polar membrane compartments, expressed Radixin and Ezrin at their canalicular-like membranes (Fig. 1B). Additionally, Radixin and Ezrin are localized in intracellular vesicles and, Ezrin more than Radixin, at the non-canalicular membrane compartments of HepaRG cells.

Since HepaRG cells have been isolated from a Hepatitis C Virus infection-associated liver tumor, depicting both hepatocyte-like and cholangiocyte-like phenotypes [34], we wanted to exclude tumor-associated Ezrin expression.

To this end, we utilized purified primary human hepatocyte donor pools from 20 female or male donors and isolated primary murine hepatocytes pooling three male or female mice to perform western blotting. We compared Ezrin and Radixin expression between those hepatocytes and HepG2, a hepatocellular tumor cell line, with previously detected Radixin and Ezrin [35] and non-parenchymal murine liver cells. As a positive control for Ezrin expression, we used murine brain tissue, given its strong Ezrin expression [36]. The results depict a strong expression of Radixin in primary human hepatocytes. Ezrin and Radixin were detected in murine brain tissue and HepG2 cells, underlining the tumor-associated Ezrin expression. Primary human hepatocytes from male donors showed a faint Ezrin band, which was not detected in the female cohort. Isolated primary hepatocytes and non-parenchymal liver cells (NPCs) from mice showed a strong Radixin expression, Ezrin was not detected in these cell populations (Fig. 1C).

In the context of Radixin expression described in the literature, our results confirmed its expression and importance in human and mouse hepatocytes. However, we detected Ezrin mainly in immortal human tumor cell lines. While the male human donor pool showed a faint Western blot band for Ezrin, we could not detect Ezrin in any of the other human and murine hepatocyte samples, nor did we detect it in murine hepatocytes using fluorescence staining. The similarity between Ezrin and Radixin, the accompanying uncertainties of antibody selectivity, and the difficulties in obtaining highly purified primary hepatocyte populations prompted further investigation of Ezrin expression. Chai et al. [26] detected Ezrin in human hepatocytes during cholestasis, while Clapéron et al. [37] found Ezrin during liver regeneration in a small pool of hepatocytes, suggesting that Ezrin expression might be intermittent in hepatocytes. To thoroughly examine the function of Ezrin in liver development, physiological function, and cholestatic disease over time, we created a hepatocyte-specific Ezrin-knockout (Ezr^ko/ko^) mouse line by crossing Ezrin-flox [30] mice with an Albumin-Cre [38] mouse line - expressing a cassette with 7 Cre-recombinases under an Albumin promoter in hepatocytes.

### 2.2 Development and liver function of hepatocyte-specific ezrin knockout mice

**Fig. 2:**
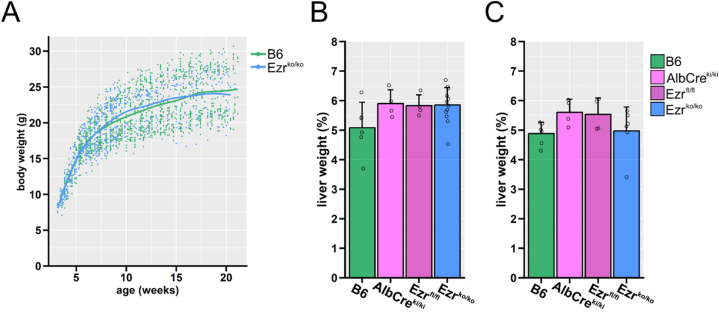
**Characterization of hepatocyte-specific Ezrin-knockout mice** **(A)** Body weight development of C57BL/6 (B6) wild-type and hepatocyte-specific Ezrin knockout (Ezr^ko/ko^) mice between 3.5 and 20 weeks after birth. The curves are computed by the local regression model “locally estimated scatterplot smoothing (LOESS)", formula = y ∼ s(x, bs = “cs”), n = 82 Ezr^ko/ko^ (36 female and 46 male); 96 B6 (51 female and 45 male). **(B, C)** Liver weight expressed relatively to body weight in B6, reference B6.Cg-Tg(Alb-cre)21Mgn/J (AlbCre^ki/ki^); B6-TG(Ezr-loxp-deltaβ-geo) (Ezr^fl/fl^), and Ezr^ko/ko^ mice. The bars indicate mean relative liver weight + SD from (B) 6 B6, 4 AlbCre^ki/ki^, 4 Ezr^fl/fl^ and 12 Ezr^ko/ko^ mice, aged 8–10 weeks, and (C) 6 B6, 4 AlbCre^ki/ki^, 4 Ezr^fl/fl^, and 6 Ezr^ko/ko^ mice, aged 12–16 weeks. Analyzing the data with an unpaired non-parametric Wilcoxon Rank Sum Test with Benjamini & Hochberg p-value adjustment method (Reference strains vs. Ezr^ko/ko^) yielded no statistically significant difference.

**Fig. 3:**
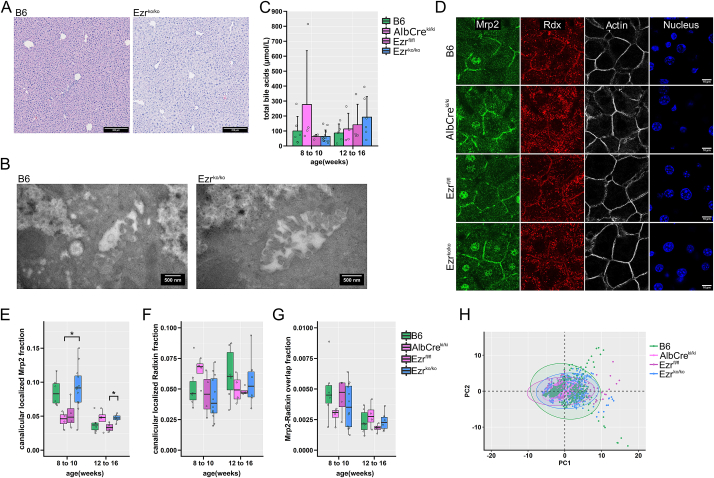
**Liver morphology and function of hepatocyte-specific Ezrin-knockout mice** **(A)** Hematoxylin eosin staining of liver tissue from naive WT (B6) and hepatocyte-specific Ezrin-knockout (Ezr^ko/ko^) mice. n = 4–12 animals/group. Scale bar 200 μm. (**B**) Representative electron micrographs of canaliculi from untreated B6 and Ezr^ko/ko^ mice. n = 3 with at least 7 analyzed canaliculi/animal. Scale bar 500 nm. (**C**) Total bile acids from homogenized liver tissue of untreated B6, reference B6.Cg-Tg(Alb-cre)21Mgn/J (AlbCre^ki/ki^); B6-TG(Ezr-loxp-deltaβ-geo) (Ezr^fl/fl^), and Ezr^ko/ko^ mice, aged 8–10 and 12–16 weeks. n = 4–12 animals/group. The bars indicate mean + SD. Data were analyzed using an unpaired non-parametric Wilcoxon Rank Sum Test with Benjamini & Hochberg p-value adjustment method, *p < 0.05 (B6, Alb-Cre^ki/ki^, Ezr^fl/fl^ vs. Ezr ^ko/ko^). (**D**) Liver cryo-sections were obtained from 8 to 16 week-old mice and stained for Mrp2, Radixin (Rdx), F-actin (Phalloidin), and Nucleus (DAPI). A representative image set from B6, Alb-Cre^ki/ki^, Ezr^fl/fl,^ and Ezr^ko/ko^ is depicted. Scale bar 10 μm. Images were taken with a confocal laser scanning microscope (LSM-780 AxioObserver, Zeiss AG, Germany), 630-fold (LD C-Apochromat 63x, NA 1.40 oil, Zeiss AG, Germany) magnification, and optimized filter settings for all dyes. n = 4–12 animals/group. (**E-G**) The Tukey Boxplots (with single data points as dots) depict key measures obtained from the automated image analysis of fluorescent Mrp2, Rdx, and F-actin imaging. The mean of the single quantified images was formed and depicted for each animal (4–12 animals/group). (**E**) Analysis of the Mrp2-fraction and (**F**) the Rdx-fraction that overlays with F-actin at the canalicular membrane in the liver tissue. (**G**) overlap of the Mrp2 and Rdx channels. The data were analyzed using an unpaired non-parametric Wilcoxon Rank Sum Test with Benjamini & Hochberg p-value adjustment method, *p < 0.05 (B6, Alb-Cre^ki/ki^, Ezr^fl/fl^ vs. Ezr ^ko/ko^) (**H**) A principal component analysis (PCA) was performed on the complete results from the automated image analysis (additional image analysis parameters Supplementary Fig. 3 A-D) for an unguided interpretation of differences between the genotypes. The principal component PC 1 and 2, inheriting the highest variance between the groups, is plotted here. Additional dimensions (PC1 vs. PC3, PC2 vs. PC3) gave similar results and are plotted in Supplementary Fig. 3 E. Each data point in the PCA plot presents an individual image. Color-matched ellipses depict the 95 % confidence areas.

At day 15 of embryonal development, the albumin promoter becomes active in mice [39] and induces expression of albumin and Cre-recombinase. Therefore, the liver-specific Ezrin knockout (Ezr^ko/ko^) is achieved during early embryonal development. Primary hepatocytes of Ezr^ko/ko^ mice were isolated and stained for Ezrin and Radixin, confirming that hepatocytes of Ezr^ko/ko^ mice did not express Ezrin (Fig. 1C). Ezr^ko/ko^ mice were born of normal appearance and developed without clinical signs of liver disease. Furthermore, the offspring number obtained from homozygous breeding was average, indicating unaffected fertility and no role of Ezrin in the early development of hepatocytes after embryonic day E15. Since liver-function-related effects were the most likely phenotypes, we analyzed body weight and liver enzymes between the different mice strains. The body weights obtained over 16 weeks from 96 B6 wild-type mice (51 female, 45 male) and 82 Ezr^ko/ko^ (36 female, 46 male) showed no difference (Fig. 2A). Further analysis of 8–16 week-old animals assumed that any difference in liver function would be fully developed and established by then. The relative liver weight in this age profile did not differ among the genetic groups, indicating stable liver growth in Ezr^ko/ko^ (Fig. 2B). The concentration of liver cell death markers aspartate aminotransferase (ASAT) and alanine aminotransferase (ALAT) measured in blood plasma was within subclinical limits (Supplementary Figs. 2A and B). As markers for hepatocyte synthetic and metabolic function, we quantified albumin and cholesterol plasma concentrations. Both showed stable concentrations and did not differ between genotypes, indicating an unimpaired liver function in Ezr^ko/ko^ mice aged 8–16 weeks (Supplementary Figs. 2C and D). The morphology was further investigated through different staining and microscopy techniques. H&E-stained liver tissue of Ezr^ko/ko^ revealed a typical liver architecture concerning hepatocyte size, number, and vascularization, without inflammatory infiltrates or apparent differences to wild-type mice (Fig. 3A). Small necrotic areas were detected in 1 of 18 Ezr^ko/ko^ and 1 of 8 Alb-Cre^ki/ki^ mice - 0.23 % and 0.72 %, respectively.

Since Ezrin may modulate the canalicular membrane and its excretory function, a set of animals were further perfused with fixative for subsequent electron microscopic tissue investigation. The canalicular membrane is characterized by a Radixin-dependent brushed border and remains unaffected by hepatocyte-specific Ezrin knockout (Fig. 3B). Total bile acids from homogenized liver tissue were analyzed by mass spectrometry to check for changes in hepatocyte excretory function. Total bile acids varied slightly between animals and groups (Fig. 3C). However, those variations were all in a subclinical range and observed across all studied genotypes. This might be due to Cre-recombinases expression, which may cause a low amount of hepatocellular stress, the individual animal's nutrition, or its activity status at the time of the experiment.

Since the immunofluorescent staining of Ezrin and Radixin in B6 mice identified an accumulation of Radixin at the canalicular membrane of hepatocytes (Fig. 1A), we examined whether the Ezr^ko/ko^ altered the localization of canalicular membrane-associated Radixin or ERM-bound Mrp2, using immunofluorescent staining (Fig. 3D). For orientation, we utilized F-actin, which has the highest density at the canalicular, followed by the other apical and basolateral membranes. Mrp2 staining accumulated at the canalicular membrane in all genetic groups. In addition, an off-target localized signal was observed at the nucleus, originating from an unspecific antibody binding or, more likely, due to the homogeneity of the nuclear staining with DAPI, which was required for image analysis.

Small intracellular Mrp2-stained vesicles were also detected. Finally, Radixin staining showed a strong signal in hepatocytes from vesicles and membranes. From an observational perspective, no differences appeared among the groups. Investigating whether that observation could be quantified, we employed cortical actin, describing the canalicular membrane as a reference for automated image quantification. If Ezrin is responsible for a partial anchoring of Mrp2 and maintenance of the canalicular pole, those effects might become visible in a quantitative approach. Thus, we calculated the fraction of canalicular localized Mrp2 (Fig. 3E) and Radixin (Rdx) (Fig. 3F) in the stained tissue based on the cortical F-actin staining. The Mrp2 and Radixin signal overlap was also quantified, excluding the false-positive nuclear Mrp2 signal based on morphological characteristics (Fig. 3G). An overall drop of Mrp2 staining and Radixin/Mrp2 overlap was observed in older animals regardless of genotype.

In those markers we considered vital for function of the canalicular membrane, no effects of a hepatocellular Ezrin knockout were apparent. With automated image analysis established, our search was extended to other staining characteristics, including total, canalicular and cytosolic Mrp2 and Radixin signals (Supplementary Figs. 3A–D). To exclude other biases due to over-interpretation of the obtained data, we performed a principal component analysis (PCA) evaluating significant changes among all immunofluorescent data obtained from B6, AlbCre^ki/ki^, Ezr^fl/fl,^ and Ezr^ko/ko^ mice. The first two principal components (PC) depicted in Fig. 3H account for 68 % of the variance and are the highest of all three analyzed (PC1 vs. PC3, PC2 vs. PC3) (Supplementary Fig. 3E). Each analyzed image from 4 to 12 animals per genotype is depicted as a dot in the PCA, and color-matched ellipses indicate respective confidence ranges. This unguided statistical analysis failed to separate Ezr^ko/ko^ from the wild-type (B6) mice and the other reference strains AlbCre^ki/ki^ and Ezr^fl/fl^, suggesting that liver-specific Ezrin knockout did not alter expression or localization of Mrp2 and Radixin in healthy 8 - 16 week-old mice.

ERM protein function as a crosslinker at the canalicular hepatocyte membrane depends upon activation via phosphorylation at EzrinT567, RadixinT564, and MoesinT558 [26,29]. As such, we used immunofluorescent imaging to examine whether the hepatocyte-specific Ezrin knockout altered ERM activation in hepatocytes. However, since the phosphorylation site responsible for activation is highly conserved between ERM proteins, the antibody we utilized could not distinguish between different ERM isoforms detecting phosphorylation at EzrinT567, RadixinT564, and MoesinT558 (pERM). pERM distributed mainly at the canalicular membrane of hepatocytes, overlapping with canalicular Radixin and F-actin in all genotypes (Supplementary Fig. 4A). We again used cortical actin to describe the canalicular membrane for automated image quantification.

Automated image quantification revealed increased canalicular localized pERM fraction and an increased Radixin and pERM overlap in older animals (Supplementary Figs. 4B and C). pERM expression and Radixin/pERM overlap did not differ between the Ezr^ko/ko^ and wild-type stains. A global PCA analysis was instigated to examine the whole immunofluorescence data set, containing data from pERM, Radixin, and F-actin staining. The principal components (PC) 1, 2, and 3 are plotted against each other, with single images as data points and color-matched ellipses representing the 95 % confidence interval (Supplementary Figs. 4D–E). PCA analysis, representing 92 % of the variance in the first three PCs, failed to separate the Ezr^ko/ko^ from reference stains B6, AlbCre^ki/ki^, and Ezr^fl/fl^, indicating that Ezrin does not contribute to activated ERM proteins in hepatocytes of healthy mice aged 8–16 weeks.

### 2.3 Hepatocyte-specific ezrin knockout's impact on obstructive cholestasis

**Fig. 4:**
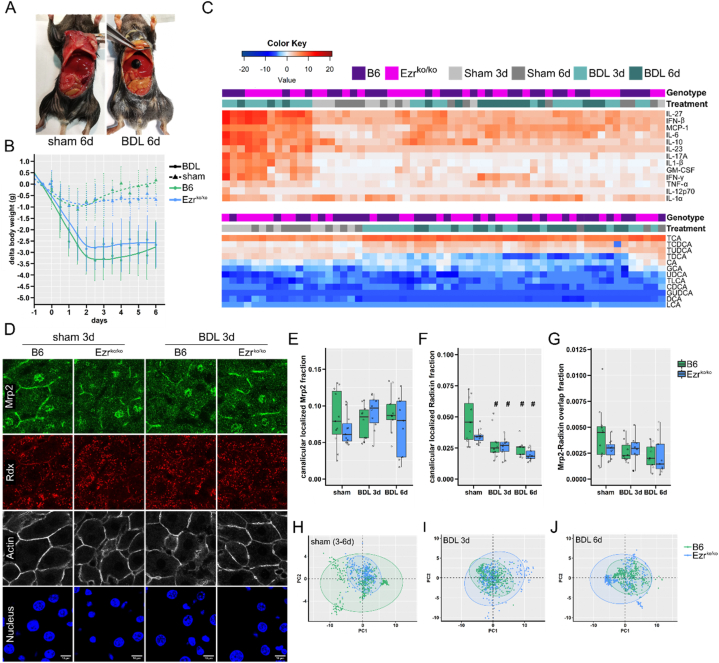
**Effect of obstructive cholestasis via BDL on wild-type vs. Ezrin knockout mice** **(A)** Autopsy of animals six days after a bile duct ligation (BDL) or sham surgery (abdominal surgery without BDL) **(B)** Body weight development in sham- and BDL-operated animals of wild-type (C57BL/6, (B6)) and hepatocyte-specific Ezrin knockout (Ezr^ko/ko^) mice (n: sham = 11, BDL 3d = 10, BDL 6d = 9). **(C)** Quantification and unguided clustering of 13 cytokines (Interleukin (IL)-27, IL-6. IL-10, IL-23, IL-17A, IL-12p70, IL-1β, IL-1α, Interferon (IFN)-β, IFN-γ, Monocyte chemoattractant protein (MCP)-1, Granulocyte-macrophage colony-stimulating factor (GM-CSF), Tumor necrosis factor (TNF)-α) quantified from plasma and 12 conjugated and unconjugated bile acids (cholic acid (CA), chenodeoxycholic acid (CDCA), deoxycholic acid (DCA), lithocholic acid (LCA), ursodeoxycholic acid (UDCA) and their taurine (T) and glycine (G) conjugates) in the liver tissue. Data were log2 transformed, and non-detected markers were set to the lower detection limit. (**D**) Liver cryo-sections were obtained from 8 to 10 week-old sham and BDL-operated animals and stained for Mrp2, Radixin (Rdx), F-actin (phalloidin), and Nucleus (DAPI). A representative image set from B6 mice and Ezr^ko/ko^ three days after surgery is shown. (**E-G**) The Tukey Boxplots (with single data points as dots) depict key measures obtained from the automated image analysis of fluorescent Mrp2, Rdx, and F-actin imaging. For each animal (n: sham = 11, BDL 3d = 10, BDL 6d = 9), the mean of the single quantified images were formed and depicted. (**E**) Analysis of the Mrp2-fraction and (**F**) the Rdx-fraction that overlays with F-actin at the canalicular membrane in the liver tissue. (**G**) overlap of the Mrp2 and Rdx channels. The data were analyzed using an unpaired non-parametric Wilcoxon Rank Sum Test with Benjamini & Hochberg p-value adjustment method, *p < 0.05 (B6 vs. Ezr ^ko/ko^) and #p < 0.05 (sham vs. BDL treatment groups). (**H-J**) Principal component analysis (PCA) was performed on the complete results from the automated image analysis (additional image analysis parameters Supplementary Fig. 6 A-D) for an unguided interpretation of differences between the genotypes. The principal component PC 1 and 2, inheriting the highest variance between the groups, is plotted here. Additional dimensions (PC1 vs. PC3, PC2 vs. PC3) gave similar results and are plotted in Supplementary Fig. 6 E, F. Each data point in the PCA plot presents an individual image. Color-matched ellipses depict the 95 % confidence areas. (**H**) sham-operated mice, (**I**) BDL 3d mice, (**J**) BDL 6d mice.

As no abnormalities were found in liver development and function of Ezr^ko/ko^ mice - using the well-established bile duct ligation (BDL) model that most similarly matches the obstructive cholestasis with previously observed Ezrin expression in humans - we focused on the potential influence of the Ezr^ko/ko^ during obstructive cholestasis [26]. Surgical bile duct ligation was performed on animals aged 8–9 weeks. Consequent effects in animals with a BDL were compared 3 or 6 days after surgery to those that received abdominal surgery without BDL (sham group) - controlling and separating effects originating from abdominal surgery and cholestasis. After BDL surgery, both Ezr^ko/ko^ and B6 control animals lost more body weight than sham-operated animals, which also recovered faster (Fig. 4B). An autopsy confirmed cholestasis, apparent by macroscopic visual jaundice and a enlarged gallbladder, in all BDL and no sham-operated animals (Fig. 4A). Clinically, the severity of cholestasis did not differ between B6 and Ezr^ko/ko^ mice.

To thoroughly examine the impact of obstructive cholestasis on the hepatocyte-specific Ezrin knockout, we began by quantifying liver cell death markers, liver synthesis markers, and a murine cytokine profile from plasma of BDL and sham-operated animals. Blood drawing from animals with progressed cholestasis at day 6 after BDL had a low efficiency due to dehydration originating from intestinal osmotic fluid loss, resulting from the missing bile acids essential for lipid uptake. Therefore, not all BDL 6d animals returned sufficient plasma to quantify liver cell death and liver synthesis markers. We measured a moderate increase in liver cell death markers ASAT and ALAT in BDL animals 3 and 6 days post-surgery. Cholesterol, metabolized in hepatocytes and excreted into the bile, was also elevated in BDL animals, Albumin, synthesized in hepatocytes, remained stable in all treatment groups. These results align with previous observations, which describe the BDL model as a cholestatic model with mild liver injury [40]. The effects of BDL-induced cholestasis on liver cell death and synthesis markers did not differ between Ezr^ko/ko^ and B6 mice (Supplementary Fig. 5). For further analysis and comparison of inflammation profiles, data obtained from the measurement of 13 cytokines were log2 transformed and submitted to unguided clustering, wherein mice with similar cytokine profiles are clustered together (Fig. 4C). The data did not cluster by genotype (Ezr^ko/ko^ vs. B6 mice), indicating that the knockout does not influence cytokine response. A group of twelve BDL animals, composed of both Ezr^ko/ko^ and B6 mice, separated from the others with elevated levels of cytokines, especially Interleukin-6, -10, −27, and Interferon (INF)-β. Thirteen sham-operated animals clustered with lower levels of cytokines, while the others mixed with the remaining BDL animals. Obstructive cholestasis, therefore, might affect cytokine response, but lack of clear separation of sham and BDL animals suggests an overlying effect from surgery. The same method was applied to the data set of 12 bile acids quantified from homogenized liver tissue. Unguided clustering separated sham from BDL animals but failed to separate the genotypes in both treatment groups. The cholestasis induced by BDL prompts an elevation of total bile acids, particularly Taurin conjugates, but simultaneously decreases unconjugated bile acids in both Ezr^ko/ko^ and B6 mice (Fig. 4C).

Previous studies reported activated Ezrin at the canalicular membrane of hepatocytes during obstructive cholestasis in humans, mediating MRP2 internalization and degeneration upon activation [26]. To check for Ezrin-mediated effects on the canalicular membrane constitution in cholestasis, we stained liver tissue obtained from Ezr^ko/ko^ and B6 mice after surgery for Mrp2 and Radixin (Fig. 4D) as well as pERM and Radixin (Supplementary Fig. 7A). As described above, F-actin was used for orientation and automated image quantification in both stainings. Automatic image quantification illustrated a slightly increased membrane distribution of Mrp2 after BDL, compared to sham-operated animals in B6 and Ezr^ko/ko^ mice. Radixin canalicular fraction was significantly reduced upon BDL, while the overlap of Mrp2 and Radixin remaining stable at day 3 showed a slight reduction at day 6 after BDL in both genotypes. No Ezr^ko/ko^ genotype-specific features were found for Mrp2 and Radixin overlap or localization. Using the previously established image quantification, we examined additional parameters of the obtained image dataset (Supplementary Fig. 6 A-D). Total Mrp2 signal was increased on day 3 after BDL, compared to sham animals, and normalized again on day 6 (Supplementary Fig. 6 A). In contrast, total Radixin signal was significantly reduced at both time points after BDL (Supplementary Fig. 6 B). Again, BDL-induced cholestasis failed to produce differences between Ezr^ko/ko^ and B6 mice. F-actin, composed of polymerized G-actin molecules, showed a continuous increase upon BDL in both genotypes, accounting for cholestasis-induced actin cytoskeleton remodeling (Supplementary Fig. 6 C).

PCA analysis was employed to complete image analysis and check for any underlining differences in the Mrp2, Radixin, and F-actin staining data set from Ezr^ko/ko^ and B6 mice. PC dimensions 1 and 2, accounting for the highest variance of all examined dimensions, are depicted with single images as data points and color-matched 95 % confidence ellipses in Fig. 4 H-J. Accounting for 74 % (Sham), 69 % (BDL3d) and 72 % (BDL6d) of variance, no separation of Ezr^ko/ko^ and B6 mice was detected in any of the treatment groups. Plotting additional dimensions (PC1 vs. PC3; PC2 vs. PC3) confirmed these findings (Supplementary Figs. 6E and F).

pERM signal, depicting the activating phosphorylation at EzrinT567, RadixinT564 and MoesinT558, was severely reduced in BDL animals. Automated image quantification confirmed this observation, showing equal canalicular localized pERM fraction reduction in both Ezr^ko/ko^ and B6 mice (Supplementary Figs. 7A and B). Simultaneously pERM and Radixin signal overlap was reduced in BDL animals regardless of their genotype (Supplementary Fig. 7C). PCA analysis was performed using the data obtained from automated image quantification of pERM, Radixin, and F-actin staining. PC dimensions 1, 2 and 3 were plotted against each other for each treatment group, with single images as data points and color-matched 95 % confidence ellipses. PCA analysis failed to separate Ezr^ko/ko^ and B6 mice in any treatment groups, indicating that Ezrin does not contribute to activated ERM proteins in hepatocytes during obstructive cholestasis (Supplementary Fig. 7D-L).

## 3 Discussion

This work presents a detailed study of Ezrin expression in hepatocytes utilizing cell models, primary cells, and knockout mice. To this day, the function of ERM proteins Ezrin and Radixin in hepatocytes is controversially discussed. Two opposing functions have been described, with activated Radixin stabilizing MRP2 at the canalicular membrane of hepatocytes, whereas activated Ezrin removes MRP2 from the membrane, promoting cholestasis [26,29].

We observed that human and murine hepatocytes were stained for Radixin, in line with previous reports. On the other hand, we found Ezrin expression in non-parenchymal liver cells and the investigated liver tumor cell lines (HepG2 and HepaRG). While HepG2 cells are mostly undifferentiated, HepaRG cells form a canalicular-like pole, where both Ezrin and Radixin are present. Notably, Ezrin is known for its oncogenic character and Ezrin overexpression has previously been associated with increased invasiveness and tumor metastasis [41,42]. Therefore, the oncogenic origin and the ability to differentiate into cholangiocyte-like cells could account for the apical Ezrin expression in HepaRG cells and explain the differing results in cell lines, tissues, and primary cells. A faint Ezrin expression was also detected in human primary hepatocytes of male donors, although we did not detect Ezrin in any of the other primary hepatocyte samples. Since it is found in non-parenchymal liver cells, such as cholangiocytes [25], contamination with these cells could account for the faint Ezrin signal in the male donor hepatocytes.

Studying Ezrin expression and function has been challenging given the remarkable homology of ERM proteins Ezrin and Radixin, difficulties isolating pure hepatocyte populations, and the lack of viable Ezrin knockout models [30,43]. Ezrin and Radixin isoforms split from a common precursor around the chordate/vertebrate boundary and are highly conserved in structure and function between mammals, rendering murine models well-suited to study ERM function on a molecular level [19,44]. Thus, to validate the absence of Ezrin in hepatocytes, we developed a liver-specific Ezrin knockout mouse and examined Ezrin's function extensively in health and cholestasis. In addition, we explored general parameters of growth, clinical appearance, and liver function in which Ezrin and Radixin may be involved, according to previous work [5,26,27]. Ezr^ko/ko^ mice grew up with no apparent differences from wild-type mice. Liver cell death and liver synthesis marker proteins of Ezr^ko/ko^ mice were within normal limits and did not differ from reference stains. Total bile acid levels varied between the different genetic and wild-type mice on a subclinical level, most likely due to natural variation of bile acids depending on the nutritional status and activity of the animals. ERM function depends on activation via phosphorylation of a conserved Threonine EzrinT567, RadixinT564, and MoesinT558. No changes in phosphorylated ERM proteins in Ezr^ko/ko^ mice were detected - quantified by immunofluorescent microscopy - suggesting that only RadixinT564 contributes to the pERM signal measured in hepatocytes of healthy mice aged 8–16 weeks. In gut epithelial cells, Ezrin is involved in microvilli formation and stabilization of MRP2 at apical membranes [30]. However, no changes in the canalicular membrane morphology or Mrp2 localization were detected in hepatocytes of Ezr^ko/ko^ mice.

Furthermore, obstructive cholestasis failed to elicit an Ezr^ko/ko^-specific liver phenotype regarding general inflammatory markers, excretory liver functions, and expression of canalicular Mrp2, phosphorylated ERM, and Radixin. Our data suggest no function for Ezrin in hepatocytes during liver development, normal liver function, or cholestasis. Thus, this study highlights Radixin as the only ERM protein expressed in hepatocytes, stabilizing proteins like MRP2 at the canalicular membrane and participating in brush border maintenance. In cholestatic diseases, changes in Radixin, but not Ezrin, activation are associated with canalicular remodeling, one of the major driving forces of cholestasis. On the other hand, Ezrin expression may be limited to non-parenchymal cells in the liver and upregulated in some tumors, promoting survival and invasiveness [45,46]. Ezrin can contribute to cholestasis in cholangiocytes, as it localizes cystic fibrosis transmembrane conductance regulator and aquaporin 1, crucial for bile fluidity and alkalinity, at the apical membrane [25]. Our data on the Ezrin function in hepatocytes suggests this as the primary mechanism for the occurrence of cholestasis described in the global Ezrin knockdown mice. In line with our results, Clapéron et al. described Ezrin expression in a small population of ductal reaction cells during liver regeneration as a sign of differentiation towards a biliary lineage, not as a general hepatocyte response to cholestasis [37]. Chai et al. utilized antibodies that specifically detect phosphorylated Ezrin in hepatocytes under cholestatic conditions in humans [26]. Since the Ezrin and Radixin phosphorylation site responsible for activation is highly conserved, it is challenging to differentiate between both isoforms by phosphorylation region [44]. The bile duct ligation model used in this study led to a loss of canalicular Radixin and antibodies detecting phosphorylated ERM proteins showed strong dephosphorylation. Meanwhile, the canalicular Mrp2 signal remained stable. Total Mrp2 increased on day 3 after BDL and Radixin Mrp2 overlap decreased slightly on day 6. These findings align with previous reports on obstructive cholestasis induced by the BDL model in rats, which found a compensatory increase in MRP2 protein levels and upregulation of anchored canalicular membrane transporters in hepatocytes [47,48]. In contrast to intrahepatic cholestasis, where the cholestatic stimulus directly affects hepatocytes, cholestatic agents in obstructive cholestasis accumulate over time. Our data suggest that removal and degeneration of canalicular Mrp2 occur after compensatory upregulation within the first 3 days after BDL. Those alterations are further presided by a drop in the phosphorylated Radixin fraction that had been described as a destabilizing event for integrated membrane transporters in the liver [29].

However, no Ezrin-dependent phenotype could be validated morphologically or functionally in the murine hepatocyte-specific Ezrin knockout model. Considering our new findings, the increased ERM phosphorylation found by Chai et al. might be due to an unspecific bound phospho-Ezrin antibody, thus showing a Radixin phosphorylation rather than Ezrin. Our results conclude that in cholestasis after initial dephosphorylation of Radixin in the onset, adaptable mechanisms may increase Radixin phosphorylation and thus traffic and anchor more transport proteins to the canalicular membrane, as a response to accumulating intracellular bile acid in hepatocytes. Future studies must examine hepatocyte Radixin activation cycles during disease progression and regeneration. Understanding how Radixin phosphorylation is managed on a cellular level and how this contributes to the formation of cholestatic disease and potential adaptational changes, will deepen understanding of the pathomechanism in excretory liver dysfunctions and cholestasis.

In summary, our study resolves a controversial question of Ezrin function in hepatocytes. We demonstrated the absence of Ezrin in hepatocytes during health and cholestasis, leaving Radixin not only as the predominant but only ERM protein expressed in hepatocytes and emphasizing its importance in organization and maintenance of the canalicular membrane structure and function.

## 4 Methods

A detailed and extended protocol for each section is provided in the Supplementary Methods.

### 4.1 Animals

Animal protocols were approved by the ethical committee and the State Agency of Thuringia, Germany (Registration No. UKJ-18-009). B6-TG(Ezr-loxp-deltaβ-geo) (Ezr^fl/fl^) mice [30] bearing a floxed Ezrin gene and B6.Cg-Tg(Alb-cre)21Mgn/J (AlbCre^ki/ki^) mice [38] (JAX #003574, Jackson Laboratory), expressing the Cre recombinase genes under an Albumin promoter were crossed generating hepatocyte-specific Ezrin knockouts (Ezr^ko/ko^). All knockout studies use animals homozygous for the floxed Ezrin Allele and heterozygous or homozygous for the Alb-Cre locus.

### 4.2 HepaRG cell culture and staining

Differentiated HepaRG cells were stained using anti-Radixin (RRID: AB_261933), and anti-Ezrin (RRID: AB_10979020) antibodies, as well as, fluorescently labeled phalloidin-DY-636 (#636-33, Dyomics GmbH, Germany) and secondary fluorescently tagged donkey-*anti*-rabbit (RRID: AB_2534017) and donkey-*anti*-mouse (RRID: AB_141607).

### 4.3 Mouse hepatocyte and non-parenchymal liver cell isolation

For each cell isolation three animals were used. Single cells were isolated from liver tissue containing non-parenchymal cells (NPC) and hepatocytes, using a single cell suspension dissociator (#DSC-400, RWD Life Science, PR China). The hepatocytes and NPCs were separated and purified using centrifugation (40 rcf, 4 min, 4 °C).

### 4.4 Western blotting

For western blotting primary human hepatocytes (PHH) and primary murine cell populations were lysed, blotted and stained with primary antibodies against Radixin (RRID: AB_2238294) and Ezrin (RRID: AB_10979020). Fluorescently tagged antibodies were used for detection (RRID: AB_10956166, RRID: AB_10953628).

### 4.5 bile duct ligation

All animals received pre- and post-surgical analgesia and fluid resuscitation and were scored in short intervals depending on their condition to provide adequate care. bile duct ligation (BDL) was performed on analgesized animals at 8–9 weeks under isoflurane anesthesia. The bile duct was ligated with 6-0 suture through a lateral incision underneath the rib cage. Animals were sacrificed to harvest tissues 3 and 6 days post-surgery.

### 4.6 Tissue histology and immunofluorescence staining

Hematoxylin and Eosin staining was performed on formalin-fixed paraffin-embedded tissue. Immunofluorescence staining was performed on frozen liver tissue. For mouse-on-mouse staining a 2 % mouse IgG-blocking antibody (Jackson ImmunoResearch, UK) was added. Primary antibodies anti-Ezrin (RRID:AB_10979020), anti-Radixin (RRID:AB_2178156), anti-MRP2 (RRID:AB_2221326), anti-Phospho-Ezrin (Thr567)/Radixin (Thr564)/Moesin (Thr558) (RRID: AB_330232) and secondary antibody donkey-*anti*-goat (RRID: AB_2534104), donkey-*anti*-mouse (RRID: AB_141607), donkey-*anti*-rabbit (RRID: AB_2535792) and 5 U mL^−1^ phalloidin-DY-636 (636-33, Dyomics GmbH, Germany) were used for staining.

### 4.7 Image analysis of fluorescence staining

Image analysis was implemented with the help of the scikit image library [49] of Python and was conducted similarly to the procedures described in Schaarschmidt et al. [50].

### 4.8 Bile acid quantification

Bile acids were quantified from homogenized liver tissue by mass spectrometry [50].

### 4.9 Electron microscopy

The liver tissue was perfused with Krebs-Henseleit Buffer (Biochrom, Germany), followed by perfusion, and embedding in fixative before imaging [50].

### 4.10 Biochemical analysis

For biochemical analysis the automated clinical chemistry analyzer (Fuji Dri-Chem 3500i, Sysmex, Germany) was used.

### 4.11 Cytokine quantification

Cytokines were quantified from EDTA plasma using the LEGENDplex Mouse Cytokine Panel (13-plex) (BioLegend, Germany) according to manufacturer's protocol.

### 4.12 Statistical analysis

Data is presented as bar blots, depicted with mean and error bars, showing the first standard deviation or as Tukey Boxplots. Single data points (animals) are shown for every blot. An unpaired non-parametric Wilcoxon Rank Sum Test with Benjamini & Hochberg p-value adjustment was applied to test for statistical differences. A p-value <0.05 was considered statistically significant.

## Funding

A doctoral scholarship from the Center of Sepsis Control and Care, Jena supported the work of Friederike Dellbrügge. The authors acknowledge the Interdisciplinary Center for Clinical Research Jena (AMSP-05) and 10.13039/501100001659Deutsche Forschungsgemeinschaft (SFB 1278, 316213987, Project C06, Z01) for funding.

## CRediT authorship contribution statement

**Friederike Dellbrügge:** Data curation, Formal analysis, Investigation, Methodology, Validation, Writing – original draft, Writing – review & editing, Project administration. **Lena D. Jesse:** Investigation, Methodology, Writing – original draft. **Anna Medyukhina:** Data curation, Formal analysis, Software, Validation, Visualization, Writing – original draft. **Na Liu:** Investigation, Methodology. **Sophie Neugebauer:** Data curation, Investigation, Methodology, Validation. **Markus Freißmuth:** Investigation, Methodology. **Stephanie Höppener:** Investigation, Methodology, Resources, Writing – original draft. **Marc T. Figge:** Funding acquisition, Project administration, Software, Supervision, Validation, Visualization, Writing – original draft. **Helen Morrison:** Conceptualization, Funding acquisition, Resources, Supervision, Validation, Visualization, Writing – original draft, Writing – review & editing. **Lars B. Riecken:** Conceptualization, Methodology, Supervision, Writing – original draft, Writing – review & editing. **Adrian T. Press:** Conceptualization, Data curation, Formal analysis, Funding acquisition, Investigation, Methodology, Project administration, Resources, Supervision, Validation, Visualization, Writing – original draft, Writing – review & editing.

## Declaration of competing interest

The authors declare that they have no known competing financial interests or personal relationships that could have appeared to influence the work reported in this paper.
