# Supplementary material for: Contribution of radixin and ezrin to the maintenance of hepatocytes' excretory function in health and disease
Source: Heliyon. 2023 Oct 18;9(11):e21009. doi: 10.1016/j.heliyon.2023.e21009 (PMC10623174; doi:10.1016/j.heliyon.2023.e21009)
Supplement: Multimedia component 1 [file mmc1.docx]

# Supplementary Information

Contribution of Radixin and Ezrin to the maintenance of hepatocytes' excretory function in health and disease.

Friederike Dellbrügge^1,2^, Lena D. Jesse^1,2^, Anna Medyukhina^3^, Na Liu^1^, Sophie Neugebauer^4^, Markus Freißmuth^1^, Stephanie Höppener^5^, Marc T. Figge^3,6^, Helen Morrison^6,7^, Lars B. Riecken^7^, Adrian T. Press^1,2,8^

1. Department of Anesthesiology and Intensive Care Medicine, Jena University Hospital, Jena, Germany
2. Center for Sepsis Control and Care, Jena University Hospital, Jena, Germany
3. Research Group Applied Systems Biology, Leibniz Institute for Natural Product Research and Infection Biology - Hans Knoell Institute, Jena, Germany
4. Department of Clinical Chemistry and Laboratory Diagnostics, Jena University Hospital, Jena, Germany
5. Laboratory of Organic and Macromolecular Chemistry (IOMC), Friedrich-Schiller University Jena, Jena, Germany
6. Faculty of Biological Sciences, Friedrich-Schiller University, Jena, Germany
7. Leibniz Institute on Aging, Jena, Germany
8. Medical Faculty, Friedrich-Schiller University, Jena, Germany

# Methods

## Animals

All animal protocols were approved by the ethical committee and the State Agency of Thuringia, Germany (Registration No. UKJ-18-009). Animals were housed in the Central Experimental Animal Facility of the Jena University Hospital (12h/12h dark/light cycles with 20 min dim phases, 21± 2 °C, humidity 55% ± 10%). B6-TG(Ezr-loxp-deltaβ-geo) (Ezr^fl/fl^) mice [30] bearing a floxed Ezrin gene were crossed with B6.Cg-Tg(Alb-cre)21Mgn/J (AlbCre^ki/ki^) mice [38] (JAX stock #003574, Jackson Laboratory), bearing approximately 7 Cre recombinase genes under an albumin promoter to generate hepatocyte-specific Ezrin knockouts (Ezr^ko/ko^). All knockout studies use animals homozygous for the floxed Ezrin Allele and heterozygous or homozygous for the Alb-Cre locus. The genotype of all genetically modified animals used in this study was confirmed before and after the experiment.

## HepaRG cell culture and staining

HepaRG cells were thawed and cultivated in William's E (#17704024, Thermo Fisher Scientific, Germany) with 10% FBS (Fetal bovine serum, Thermo Fisher Scientific, Germany) and 1% Penicillin (PanBiotech, Germany) and Streptomycin (PanBiotech, Germany) at 37°C, 90% humidity, and 5% CO_2_ for two weeks, with medium changes twice weekly. The cell layer is washed twice with HBSS (Hank's Balanced Salt Solution, #14175095, Thermo Fisher Scientific, Germany) and incubated with 1% trypsin at 37°C for 10 minutes for passaging. For the experiments, 50,000 undifferentiated HepaRG cells were seeded into each well of 8-well µ-slides with 1.5H glass bottom (#80827, ibidi, Germany) and cultivated for 2 weeks, with medium changes every other day. After 2 weeks, the medium was supplemented with 1% v/v DMSO (Carl Roth, Germany) for a week, initiating differentiation. Finally, the DMSO concentration was increased to 2% v/v for the following 2 weeks, completely polarizing the HepaRG cells before the experiments were conducted.

The staining was carried out in the µ-slide (ibidi, Germany) in a volume of at least 250 µL per well. Cells were washed with Tris Buffered Saline (TBS) (PanBiotech, Germany), before Paraformaldehyde (Carl Roth, Germany), dissolved in TBS at a concentration of 4% (w/v), was added for 5 min to each well. The cell membranes were permeabilized with TBS containing 0.5% Triton-X100 (#85111, ThermoFisher Scientific, Germany) for 30 minutes at room temperature. Any remaining permeabilization buffer was removed by carefully washing the wells 3 times with TBS, before blocking them with 10% bovine serum albumin (BSA, #3737.3, CarlRoth, Germany) in TBS for 1 hour. Next, the cells were incubated simultaneously with primary antibodies diluted 1:200 in TBS against Radixin (polyclonal rabbit-anti-Radixin, R3653, Sigma-Aldrich, Germany, RRID: AB_261933) and Ezrin (monoclonal mouse-anti-Ezrin, MA5-13862, ThermoFisher Scientific, German, RRID: AB_10979020), overnight at 4°C. After three washing steps in TBS containing 1% BSA, a mix of second antibodies and fluorescently labeled phalloidin was added, containing donkey-anti-rabbit IgG AlexaFluor 568 (A10042, ThermoFisher Germany; diluted 1:500), donkey-anti-mouse IgG AlexaFluor 488 (A21202, ThermoFisher Scientific, Germany; diluted 1:500), and 12.5 U mL^-1^ phalloidin-DY-636 (#636-33, Dyomics GmbH, Germany) for 3 h at ambient temperature. Between both and after the double staining, cells were washed again carefully 3 times with TBS. Nuclei were stained with DAPI (2 µg mL^-1^ in Phosphate Buffered Saline (PBS) (Carl Roth, Germany)) for 15 minutes at room temperature, before samples were sealed with ROTI-mount (HP68.1, Carl Roth, Germany) and stored at 4 °C in the dark until imaging.

## Laser Scanning Microscopy

The samples were analyzed on a confocal laser scanning microscope (LSM-780 AxioObserver, Zeiss AG, Germany). Images were taken at 200-fold (PlanApochromat 20x, NA 0.8 air, Zeiss AG, Germany) and 630-fold (LD C-Apochromat 63x, NA 1.40 oil, Zeiss AG, Germany) magnification, and optimized filter settings for all dyes.

## Mouse hepatocyte and non-parenchymal liver cell isolation

Animals were euthanized by an overdose of veterinarian Ketamin and Xylzin based on their body weight before laparotomy. The liver was excised and chopped into small pieces. Tissue pieces from three mice of the same sex and genotype were pooled and placed into a 50-mL tissue processing tube (#SCT-25, RWD Life Science, PR China) containing ice-cold PBS (#P04-36500, PAN-Biotech, Germany) with 5 mmol L^-1^ EDTA (#AM9260G, Invitrogen, USA), and mounted on a single cell suspension dissociator (#DSC-400, RWD Life Science, PR China). The cells were isolated by applying two dissociation protocols, the first of which required a total time of 16 s configured as follows: soft 200 for 1 sec, spin 200 for 4 sec, spin -200 for 2 sec, soft 200 for 1 sec, spin 200 for 7 sec, and spin 20 for 1 sec. Following a short visual inspection of the suspension for inhomogeneities, a second program was started, lasting 25 s in total: soft 200 for 1 sec, spin 200 for 4 sec, spin -200 for 2 sec, soft 200 for 1 sec, spin 200 for 8 sec, spin -200 for 2 sec, soft 200 for 1 sec, spin 200 for 5 sec and spin 20 for 1 sec. The single-cell suspension obtained contained the non-parenchymal cells (NPC) and hepatocytes. The homogenate was strained through a 70 µm cell strainer (#431751, Corning, USA) into a 50 mL centrifuge tube containing 15 mL ice-cold PBS with 5 mmol L^-1^ EDTA. Centrifugation of the liver suspension was utilized (40 rcf, 4 min, 4 °C) to accumulate hepatocytes in the pellet. Next, the supernatant containing NPCs was transferred to a new 15 mL conical tube, and the pellet of hepatocytes was resuspended in ice-cold PBS with 5 mmol L^-1^ EDTA and repeatedly centrifugated (40 rcf, 4 min, 4 °C) to purify the hepatocytes. The supernatant from the first centrifugation was centrifuged (500 rcf, 5 min, 4 °C) twice to isolate the pellets of NPCs. Pellets of hepatocytes and NPCs were frozen at -80 °C.

## Western blotting

Primary human hepatocytes (PHH) were purchased from Lonza, Switzerland. Lonza has all the necessary permissions to isolate and distribute these cells for research. Primary murine cell populations were obtained from mice post-mortem. Cells were lysed with RIPA buffer (50 mM TRIS-HCl, 150 mM NaCl, 1% Triton-X100, 0,5% Deoxycholate acid, 0,1% SDS, pH=7,6 with Protease Inhibitor). Lysates were cleared by centrifuging at 14,000 rcf for 15 minutes at 4 °C. Samples were subjected to SDS-PAGE on SERVAGel™ Neutral HSE precast gels (#43245.01, SERVA, Germany) before transfer to Immobilon®-FL PVDF membrane (#IPFL85R, Merck Millipore, Germany) using semidry blotting. Membranes were blocked for 1 hour at room temperature in Blocker™ FL Fluorescent Blocking Buffer (#37565, Thermo Fisher Scientific, Germany) and subsequently incubated with primary antibodies (1:1000 dilution in Blocking Buffer, monoclonal rabbit-anti-Radixin (Clone C4G4, #2636S, Cell Signaling Technology, USA, RRID: AB_2238294), monoclonal mouse-anti-Ezrin (MA5-13862, Thermo Fisher Scientific, Germany, RRID: AB_10979020)) at 4 °C overnight. After washing 3 times with TBS-Tween, membranes were incubated in secondary antibodies (1:10000 dilution in Blocking Buffer, IRDye® 680RD goat-anti-rabbit IgG (H + L)(#926-68071, LICOR, USA, RRID: AB_10956166), IRDye® 680RD donkey-anti-mouse IgG (H + L)(#926-68072, LICOR, USA, RRID: AB_10953628)) for 1 hour at room temperature. Membranes were then washed 3 times in TBS-Tween and 2x in Milli-Q water before imaging fluorescent bands with a LICOR Odyssey XF Imaging System.

## Bile Duct Ligation

Animals (male and female) were subjected to bile duct ligation surgery at the age of 8 - 9 weeks. 24 h and 1 h before surgery, all received meloxicam (Melosus (0.5 mg mL^‑1^ oral suspension), CP-Pharma, Germany) (1 mg kg^-1^ body weight (BW)). The surgery was performed under general isoflurane anesthesia on a temperature-controlled surgical table in a semi-sterile environment, with tip-sterilized surgical instruments. The abdomen of anesthetized animals was shaved, disinfected, and the bile duct ligated twice with 6-0 non-resorbable suture through a 1.5 cm long lateral incision underneath the rib cage. A second scientist validated the ligation, following a four-eye principle to minimize error rates. After the incision was sutured up (4-0 monopile, non-resorbable suture), bupivacaine (Bucain 2,5 mg mL^-1^, PUREN Pharma, Germany) was injected subcutaneously along the suture line (total 2 to 4 mg kg^-1^ BW). All animals received Ringer Acetate (Berlin-Chemie, Germany) (20 mL KG^-1^ BW) injection (s.c.) before the anesthesia ended. Until animals reached total activity, they were placed on a temperature mat with food and water ad libitum. Sham-operated animals underwent a similar surgical procedure without ligating the bile duct. Postoperative subjects were scored three times a day and bodyweight measured twice per day. Fluid resuscitation with 600 µL was continued 3 times daily for all animals.

All received meloxicam (0.5 mg kg^-1^ BW, p.o.) twice daily for the first three postoperative days. Animals were then sacrificed to harvest tissues 3 and 6 days post-surgery. Blood was withdrawn from the heart under general anesthesia and analgesia to generate EDTA plasma. Organs were weighed, snap-frozen, or fixed in 4% formalin (Roti-Histofix, Carl Roth GmbH, Germany) and paraffin-embedded.

## Tissue histology and immunofluorescence staining

Hematoxylin and Eosin staining was performed on formalin-fixed paraffin-embedded tissue. For immunofluorescence staining, fresh frozen liver tissue was fixed for 30 min (Roti-Histofix 4%, Carl Roth, Germany), then rinsed with TBS, permeabilized in 0.5% Triton-X100 in TBS for 1 h and blocked in TBS containing 10% BSA. For mouse-on-mouse staining (Figure 1), a 2% mouse IgG blocking antibody (Jackson ImmunoResearch, UK) was added to the blocking buffer. Primary antibodies (monoclonal mouse-anti-Ezrin (MA5-13862, ThermoFisher Scientific, Germany, RRID:AB_10979020), polyclonal goat-anti-Radixin (EB07306, Everest Biotech Ltd, UK, RRID:AB_2178156), polyclonal rabbit-anti-MRP2 (M8316, Sigma Aldrich, USA, RRID:AB_2221326), polyclonal rabbit-anti-Phospho-Ezrin (Thr567)/Radixin (Thr564)/Moesin (Thr558) (pERM) (#3141L, Cell Signaling Technology, USA, RRID: AB_330232)) were diluted 1:200 in blocking buffer and incubated with the tissue at room temperature for 1 h. Slides were rinsed thoroughly with TBS and incubated in secondary antibody (Alexa Fluor 568 donkey-anti-goat (A11057, Thermo Fisher Scientific, USA, RRID: AB_2534104), Alexa Fluor 488 donkey-anti-mouse (A21202, Thermo Fisher Scientific, USA, RRID: AB_141607), Alexa Fluor 488 donkey-anti-rabbit (A-21206 Thermo Fisher Scientific, USA, RRID: AB_2535792) diluted 1:200 in blocking buffer containing 5 U mL^-1^ phalloidin-DY-636 (636-33, Dyomics GmbH, Germany) for 1 h at room temperature. The slides were then washed with TBS and incubated for 5 min with 10 µg mL^-1^ Hoechst 33342 (Sigma Aldrich, Germany) in deionized water. After again washing with TBS, the tissue was mounted with Roti-Mount FluoroCare (Carl Roth, Germany). The samples were analyzed on a confocal laser scanning microscope (LSM-780 AxioObserver, Zeiss AG, Germany) with optimized settings and magnification as described above.

## Image Analysis of fluorescence staining

Image analysis was implemented with the help of the scikit image library [49] of Python and was conducted similarly to the procedures described in Schaarschmidt et al. [50]. The images were pre-processed by a median ﬁlter (size 3 px) and separated into background and foreground. To identify the background, each channel (Mrp2, pERM, Radixin, F-actin, or DAPI) was smoothed with a Gaussian filter (σ = 1.5 µm) and thresholded via a two-step Otsu threshold. The Otsu threshold was first applied to the entire image and then the lower-intensity part of the thresholded image was used to compute another Otsu threshold for the final thresholding. The results from all three channels were combined into the final background image, whereby the background included the pixels identified as the background in all three channels. Lastly, the final background image was post-processed by morphological opening and closing to filter out small background and foreground fragments.

To segment nuclei regions, the corresponding channel was smoothed with a Gaussian filter (σ = 0.5 µm) and thresholded with the Otsu threshold. To segment the F-actin, Radixin, pERM and Mrp2 positive regions, first the tissue's base level of fluorescence was estimated by computing the corresponding channel's mean intensity in the foreground area. Next, the image was thresholded at an intensity value three times higher than the base fluorescence level and then set to background those pixels belonging to the nuclei mask. The canaliculi regions were assigned to those positive pixels in the Mrp2 or pERM channels (depending on the applied staining). Finally, the cytoplasm region was assigned to those pixels that did not belong to the background, nuclei or canaliculi.

After segmentation, the area fraction of the nuclei, canaliculi and cytoplasm was computed relative to the foreground area, before the average of all other channels' and integrated intensities was computed over the foreground regions, nuclei, canaliculi, and cytoplasm. In addition, the areas of the F-actin and Radixin positive regions were quantified and normalized by the total foreground area. Next, colocalization for each pair of channels (Mrp2 or pERM, F-actin, Radixin) was quantified by the overlap area, computed as the number of double-positive pixels normalized by the total foreground area.

## Bile acid quantification

Twelve conjugated and unconjugated bile acids (cholic acid (CA), chenodeoxycholic acid (CDCA), deoxycholic acid (DCA), lithocholic acid (LCA), ursodeoxycholic acid (UDCA) and their taurine (T) and glycine (G) conjugates) were quantified from homogenized liver tissue by mass spectrometry. [50] Total bileacids were calculated by summing up the measured bile acids.

## Electron Microscopy

The liver tissue was perfused with Krebs-Henseleit Buffer (Biochrom, Germany), followed by perfusion and embedding in fixative and imaging as described before. [50]

## Cytokine Quantification

Cytokines were quantified from EDTA plasma using the LEGENDplex Mouse Th Cytokine Panel (13-plex) (BioLegend, Germany). A BD Accuri (BD Bioscience, Germany) was used per to the manufacturer's protocol to measure samples, which were analyzed against a standard curve.

## Biochemical analysis

For plasma, the collected EDTA-plasma was centrifuged at 1000 rcf for 10 min at 4°C and stored at -80°C for later analyses. The activity of liver enzymes alanine aminotransferase (ALAT) and aspartate aminotransferase (ASAT), albumin, and cholesterol were analyzed using the automated clinical chemistry analyzer (Fuji Dri-Chem 3500i, Sysmex, Germany).

## Statistical analysis

Bar blots are depicted with mean and error bars, showing the first standard deviation. Box blots are shown with median, upper and lower quartile, and whiskers. Whiskers include data minimum and maximum but at most 1.5x the interquartile interval. Single data points are shown for every blot. An unpaired non-parametric Wilcoxon Rank Sum Test with Benjamini & Hochberg p-value adjustment was applied to test for statistical differences. A p-value < 0.05 was considered statistically significant.


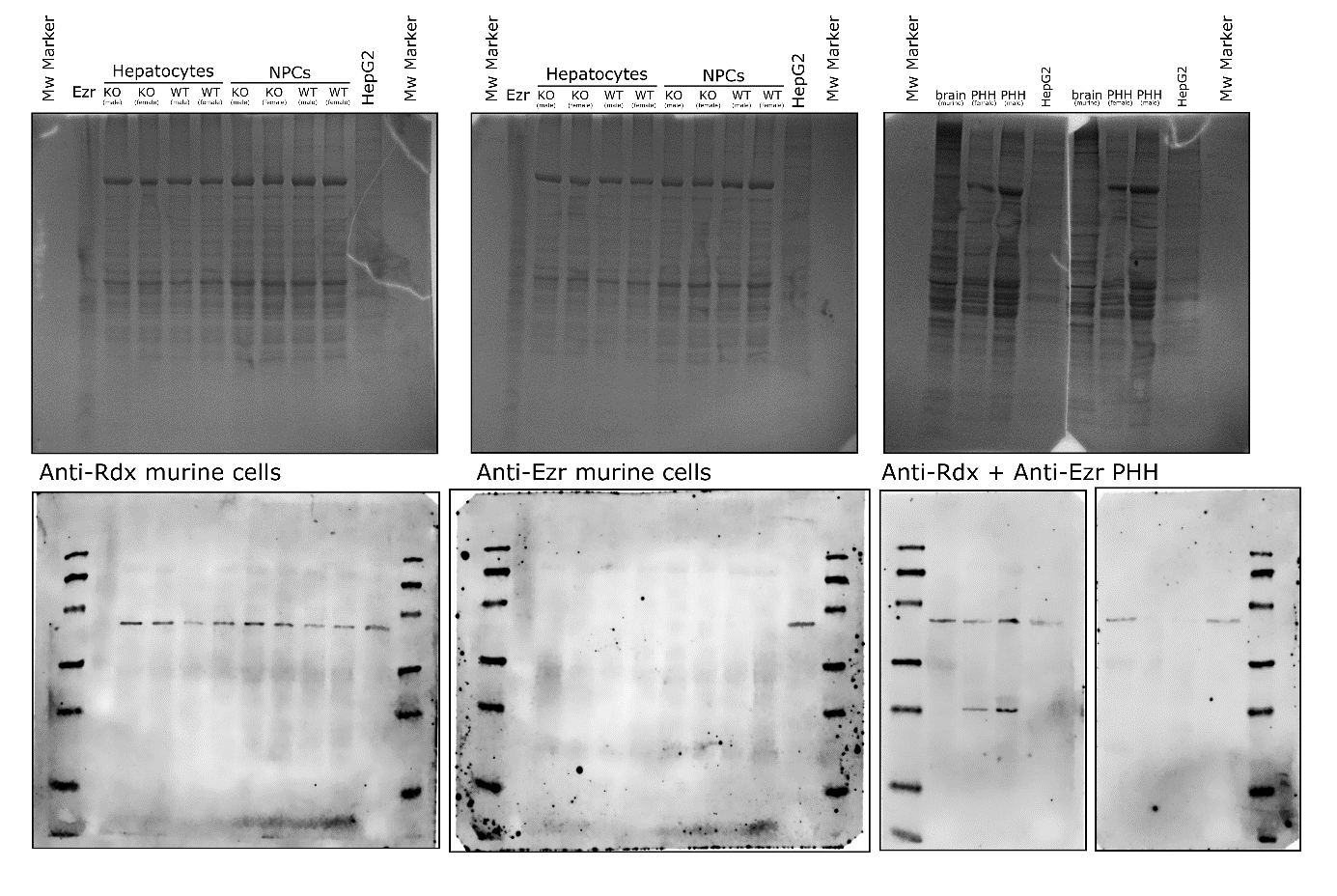


Supplementary Figure 1 Gels and membranes from western blotting of primary human and murine cells

Gel electrophoresis gels of primary hepatocytes and non-parenchymal cells (NPCs) from C57BL/6 (WT) wild-type and hepatocyte-specific Ezrin knockout (KO) mice and HepG2 cells. Gel electrophoresis gels of primary human hepatocytes (PHH) from 20 male or female donor pools, murine brain tissue, and HepG2 cells. Corresponding PVDF membranes were stained for Ezrin (Ezr) and Radixin (Rdx) and detected at 700 nm. The Chameleon® Duo Pre-stained Protein Ladder (LICOR) was used as a molecular weight marker (Mw Marker).


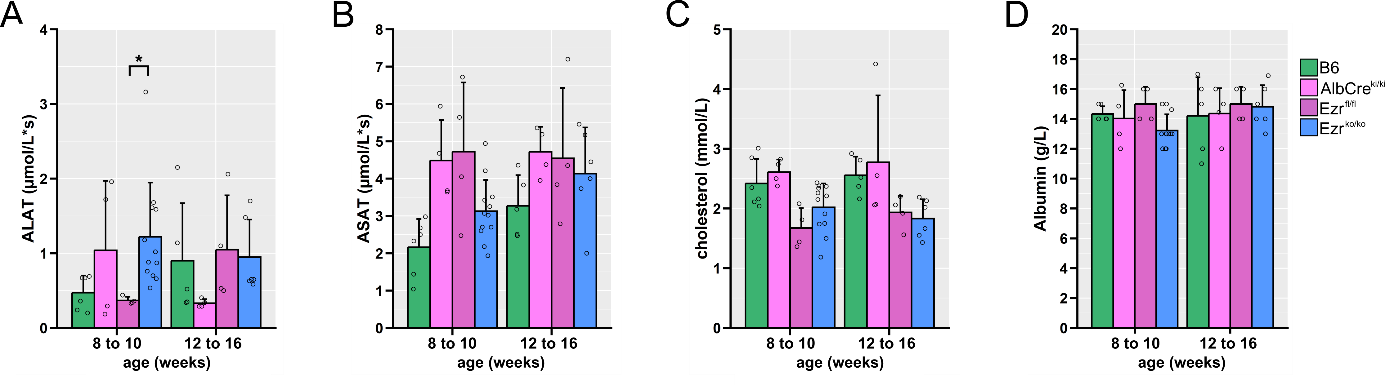


Supplementary Figure 2 Liver enzymes and liver synthesis marker in hepatocyte-specific Ezrin knockout mice

**(A-D)** Quantified from plasma of WT (B6), reference B6.Cg-Tg(Alb-cre)21Mgn/J (AlbCre^ki/ki^); B6-TG(Ezr-loxp-deltaβ-geo) (Ezr^fl/fl^), and hepatocyte-specific Ezrin knockout (Ezr^ko/ko^) mice, aged 8-10 (6 B6, 4 AlbCre^ki/ki^, 4 Ezr^fl/fl^, 12 Ezr^ko/ko^) and 12-16 (5 B6, 4 AlbCre^ki/ki^, 4 Ezr^fl/fl^, 6 Ezr^ko/ko^) weeks. The bars indicate mean + SD. The data were analyzed using an unpaired non-parametric Wilcoxon Rank Sum Test with Benjamini & Hochberg p-value adjustment method (Reference strains vs. Ezr^ko/ko^), *p < 0.05.


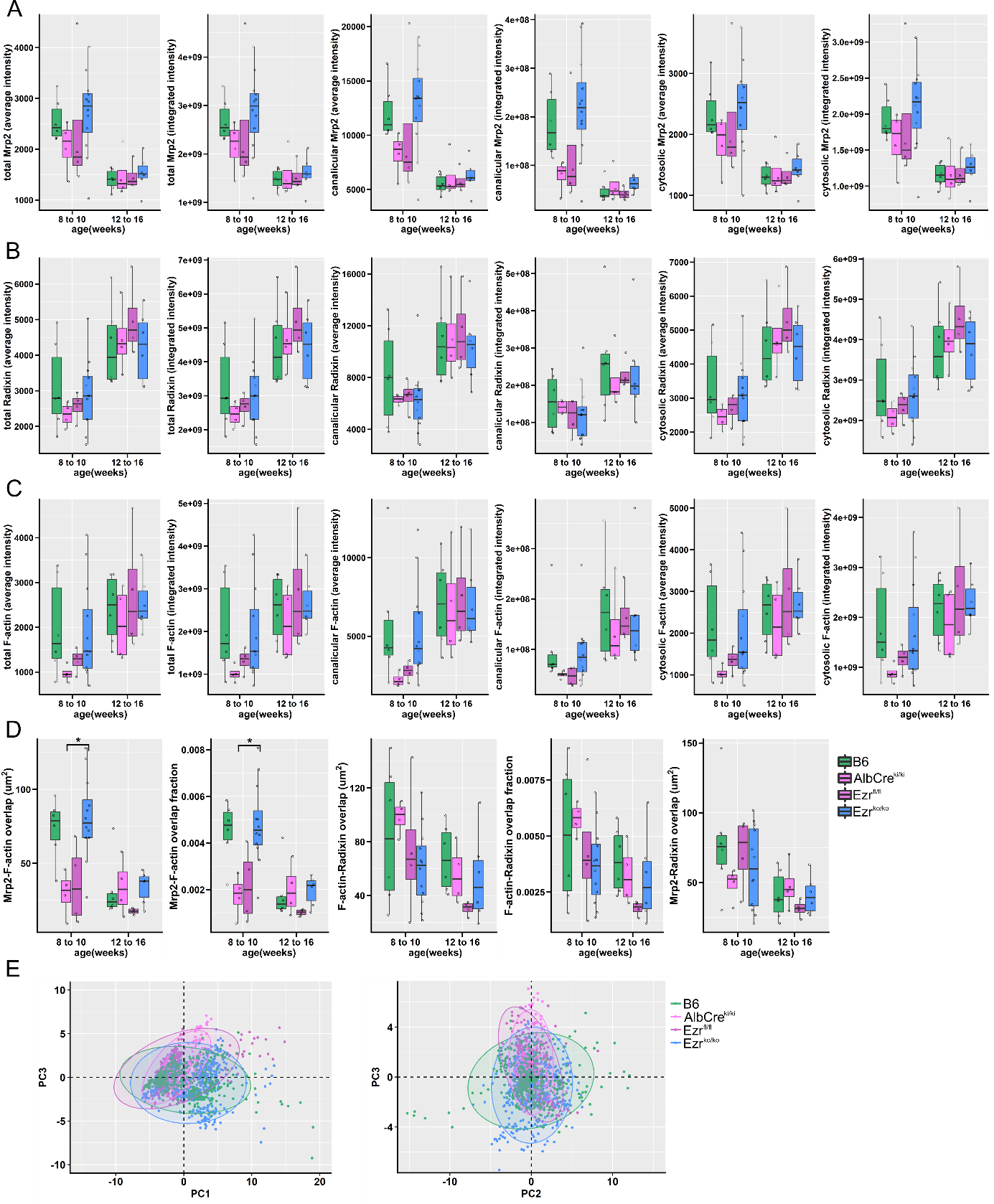


Supplementary Figure 3 Mrp2, Radixin and F-actin fluorescent image analysis of untreated mice

Liver cryo-sections were obtained from 8 - 10 and 12 - 16 week-old WT (B6), reference B6.Cg-Tg(Alb-cre)21Mgn/J (AlbCre^ki/ki^); B6-TG(Ezr-loxp-deltaβ-geo) (Ezr^fl/fl^), and hepatocyte-specific Ezrin knockout (Ezr^ko/ko^) mice and stained for Mrp2, Radixin (Rdx), F-actin (Phalloidin) and Nucleus (DAPI). Images were taken with a confocal laser scanning microscope (LSM-780 AxioObserver, Zeiss AG, Germany), 630-fold (LD C-Apochromat 63x, NA 1.40 oil, Zeiss AG, Germany) magnification and optimized filter settings for all dyes. (A-D) The Tukey Boxplots (with single data points as dots) depict measures obtained from the automated image analysis of fluorescent Mrp2, Rdx and F-actin imaging. The mean of the single quantified images was formed and depicted for each individual animal (4-12 animals/group). The data were analyzed using an unpaired non-parametric Wilcoxon Rank Sum Test with Benjamini & Hochberg p-value adjustment method (Reference strains vs. Ezr^ko/ko^), *p < 0.05. (E) Principal component analysis (PCA) was performed on the complete results from the automated image analysis. Each data point in the PCA plot presents an individual image. Color-matched ellipses depict the 95 % confidence areas. Additional dimensions (PC1 vs. PC3, PC2 vs. PC3) are plotted.


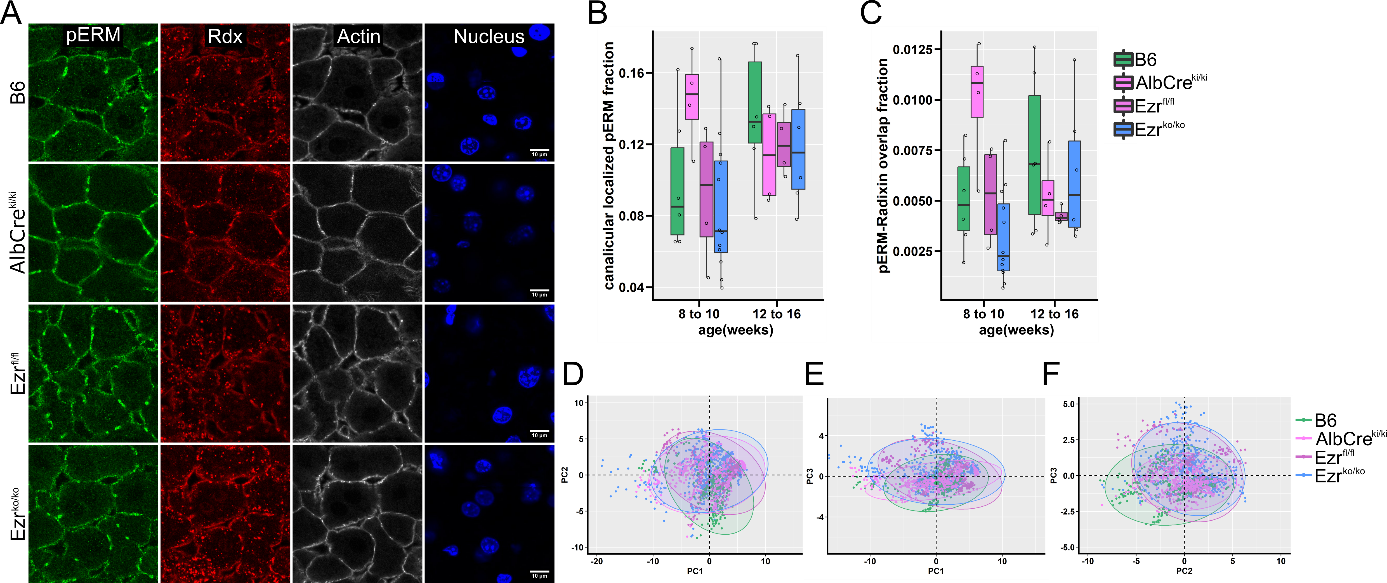


Supplementary Figure 4 ERM activation in liver tissue of hepatocyte-specific Ezrin knockout mice

Liver cryo-sections were obtained from 8 - 16 week-old WT (B6), reference B6.Cg-Tg(Alb-cre)21Mgn/J (AlbCre^ki/ki^); B6-TG(Ezr-loxp-deltaβ-geo) (Ezr^fl/fl^), and hepatocyte-specific Ezrin knockout (Ezr^ko/ko^) mice and stained for phosphorylated EzrinT567, RadixinT564, MoesinT558 (pERM), Radixin (Rdx), F-actin (Phalloidin) and Nucleus (DAPI). **(A)** A representative image set from B6, Alb-Cre^ki/ki^, Ezr^fl/fl^, and Ezr^ko/ko^ is depicted. Scale bar 10 µm. Images were taken with a confocal laser scanning microscope (LSM-780 AxioObserver, Zeiss AG, Germany), 630-fold (LD C-Apochromat 63x, NA 1.40 oil, Zeiss AG, Germany) magnification and optimized filter settings for all dyes. **(B, C)** The Tukey Boxplots (with single data points as dots) depict key measures obtained from the automated image analysis of fluorescent pERM, Rdx and F-actin imaging. The mean of the single quantified images was formed and depicted for each individual animal (4-12 animals/group). **(B)** pERM-fraction that overlays with F-actin at the canalicular membrane in the liver tissue and **(C)** overlap of the pERM and Rdx channels (canalicular Rdx-fraction is depicted in **Figure 3F**). The data were analyzed using an unpaired non-parametric Wilcoxon Rank Sum Test with Benjamini & Hochberg p-value adjustment method (Reference strains vs. Ezr^ko/ko^), *p < 0.05. **(D)** A principal component analysis (PCA) was performed on the complete results from the automated image analysis for an unguided interpretation of differences between the genotypes. The principal components (PC) 1, 2 and additional dimensions (PC1 vs. PC3, PC2 vs. PC3) are plotted here. Each data point in the PCA plot presents an individual image. Color-matched ellipses depict the 95 % confidence areas.


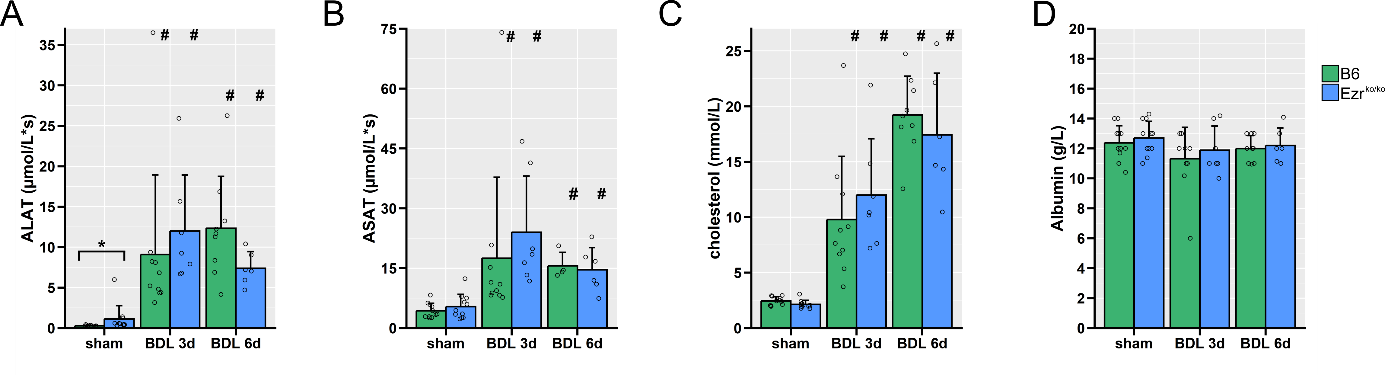


Supplementary Figure 5 Liver enzymes and liver synthesis marker after BDL of wild-type and Ezrin knockout mice

**(A-D)** Quantified from plasma of WT (B6) and hepatocyte-specific Ezrin-knockout (Ezr^ko/ko^) mice, 3 and 6 days after a bile duct ligation (BDL) or sham surgery (abdominal surgery without BDL). The bars indicate mean + SD. The data were analyzed using an unpaired non-parametric Wilcoxon Rank Sum Test with Benjamini & Hochberg p-value adjustment method. *p < 0.05 (B6 vs. Ezr ^ko/ko^) and #p < 0.05 (sham vs. BDL treatment groups).


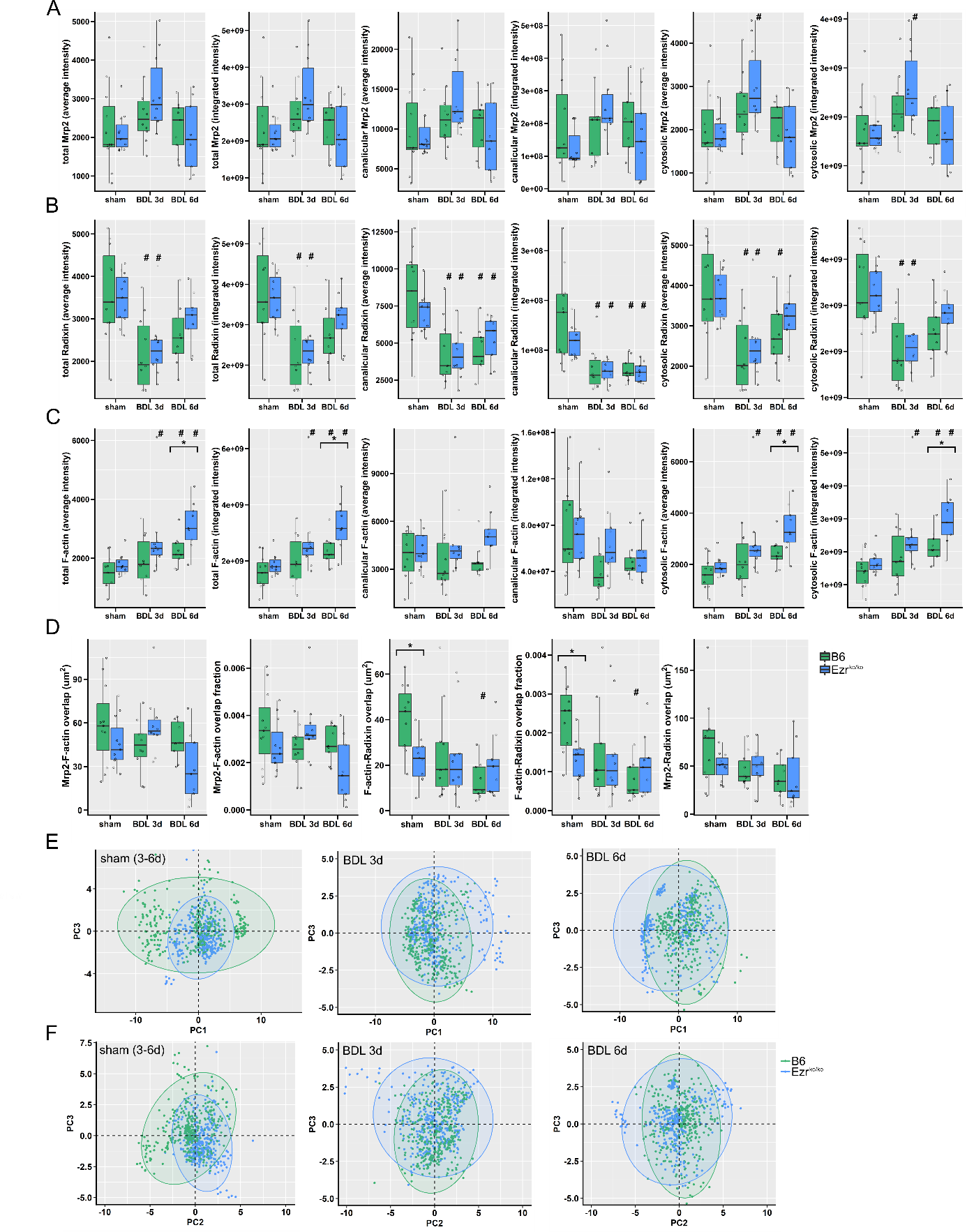


Supplementary Figure 6 Mrp2, Radixin and F-actin fluorescent image analysis of BDL and sham mice

Liver cryo-sections were obtained from WT (B6) and hepatocyte-specific Ezrin-knockout (Ezr^ko/ko^) mice, 3 and 6 days after a bile duct ligation (BDL) or sham surgery (abdominal surgery without BDL) and stained for Mrp2, Radixin (Rdx), F-actin (Phalloidin) and Nucleus (DAPI). Images were taken with a confocal laser scanning microscope (LSM-780 AxioObserver, Zeiss AG, Germany), 630-fold (LD C-Apochromat 63x, NA 1.40 oil, Zeiss AG, Germany) magnification, and optimized filter settings for all dyes. (A-D) The Tukey Boxplots (with single data points as dots) depict measures obtained from the automated image analysis of fluorescent Mrp2, Rdx and F-actin imaging. For each animal (n: sham = 11, BDL 3d = 10, BDL 6d= 9), the mean of the single quantified images was formed and depicted. The data were analyzed using an unpaired non-parametric Wilcoxon Rank Sum Test with Benjamini & Hochberg p-value adjustment method. *p < 0.05 (B6 vs. Ezr ^ko/ko^) and #p < 0.05 (sham vs. BDL treatment groups). (E, F) Principal component analysis (PCA) was performed on the complete results from the automated image analysis. Each data point in the PCA plot presents an individual image. Color-matched ellipses depict the 95 % confidence areas. Additional dimensions (E) PC1 vs. PC3 and (F) PC2 vs. PC3.


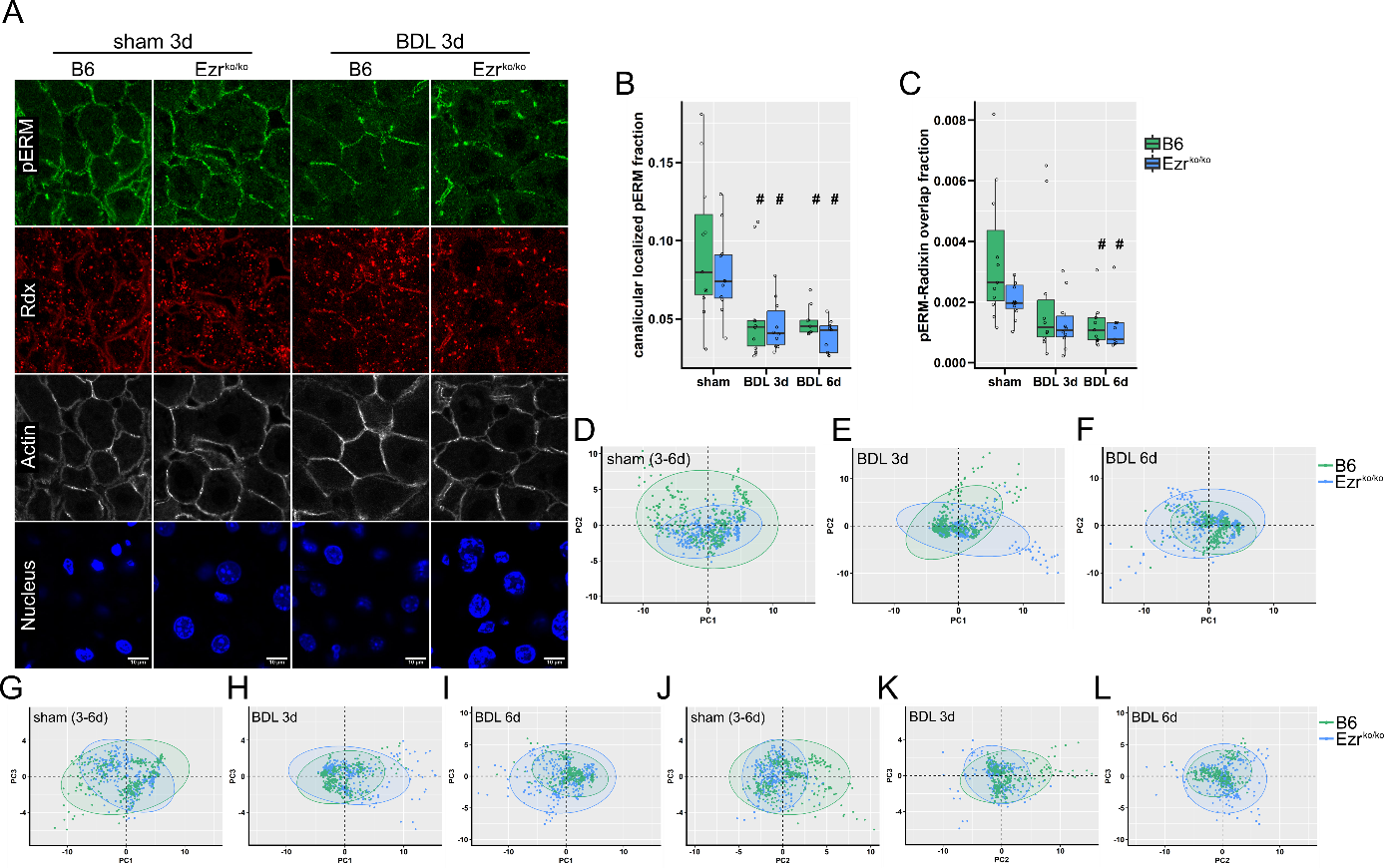


Supplementary Figure 7 ERM activation in liver tissue after BDL of wild-type and Ezrin knockout mice

Liver cryo-sections were obtained from WT (B6) and hepatocyte-specific Ezrin-knockout (Ezr^ko/ko^) mice 3 and 6 days after a bile duct ligation (BDL) or sham surgery (abdominal surgery without BDL) and stained for phosphorylated EzrinT567, RadixinT564, MoesinT558 (pERM), Radixin (Rdx), F-actin (Phalloidin) and Nucleus (DAPI). **(A)** A representative image from B6 and Ezr^ko/ko^ is depicted. Scale bar 10 µm **(B, C)** The Tukey Boxplots (with single data points as dots) depict key measures obtained from the automated image analysis of fluorescent pERM, Rdx and F-actin imaging. For each animal (n: sham = 11, BDL 3d = 10, BDL 6d= 9), the mean of the single quantified images was formed and depicted. Images were taken with a confocal laser scanning microscope (LSM-780 AxioObserver, Zeiss AG, Germany), 630-fold (LD C-Apochromat 63x, NA 1.40 oil, Zeiss AG, Germany) magnification and optimized filter settings for all dyes. **(B)** pERM-fraction that overlays with F-actin at the canalicular membrane in the liver tissue and **(C)** overlap of the pERM and Rdx channels (canalicular Rdx-fraction is depicted in **Figure 4F**). The data were analyzed using an unpaired non-parametric Wilcoxon Rank Sum Test with Benjamini & Hochberg p-value adjustment method. *p < 0.05 (B6 vs. Ezr^ko/ko^) and #p < 0.05 (sham vs. BDL treatment groups). **(D-L)** A principal component analysis (PCA) was performed on the complete results from the automated image analysis for an unguided interpretation of differences between the genotypes. The principal components PC 1 and 2 for **(D)** sham, **(E)** BDL 3d, **(F)** BDL 6d, and **(G-L)** additional dimensions (PC1 vs. PC3, PC2 vs. PC3) are plotted here. Each data point in the PCA plot presents an individual image. Color-matched ellipses depict the 95 % confidence areas.
